# A geometric morphometrics approach to sex estimation of infants from 0 to 6 years using the auricular surface

**DOI:** 10.1038/s41598-026-35321-y

**Published:** 2026-02-28

**Authors:** Patrícia Simão, Susana J. Garcia, Ricardo Miguel Godinho

**Affiliations:** 1 Interdisciplinary Center for Archaeology and the Evolution of Human Behaviour (ICArEHB), Faculdade das Ciências Humanas e Sociais, Universidade do Algarve (UAlg), Campus de Gambelas, Faro, 8005-139 Portugal; 2 Centro de Administração e Políticas Públicas (CAPP), Instituto Superior de Ciências Sociais e Políticas, Museu Nacional de História Natural e da Ciência, Universidade de Lisboa, Rua Almerinda Lessa, Lisboa, 1300-663 Portugal

**Keywords:** Virtual anthropology, Ilium, Bioanthropology, Bioarcheology, Paleodemography., Anatomy, Developmental biology, Evolution, Zoology

## Abstract

Estimating biological sex is an essential tool for studying past population dynamics and cultural behaviour. Typically, it is not performed in non-adults before the onset of puberty, limiting the knowledge that can be gained from immature skeletal remains. This study used three-dimensional (3D) geometric morphometrics (GM) to examine sexual dimorphism and assess its effectiveness in estimating biological sex in infants aged 0–6 years using the auricular surface of the ilium. This approach was tested with 46 individuals (20 females and 26 males) from the Lisbon Identified Skeletal Collection. All specimens were digitised, and standard GM analysis was conducted. Apparent sex-related morphological differences in the auricular surface were observed in infants under one year, although these did not reach statistical significance at α = 0.05. Such differences were not identified in older individuals. These findings suggest that the auricular surface may potentially serve as a sex indicator in infants under one year, whereas sexual dimorphism appears to decrease in older individuals. This study demonstrates the potential of employing 3D GM for sex estimation in non-adult skeletal remains. Further research should explore additional iliac features for infant sex estimation, incorporate larger and more diverse samples, and potentially include Artificial Intelligence techniques.

**Supplementary Information:**

The online version contains supplementary material available at 10.1038/s41598-026-35321-y.

## Introduction

Estimating biological sex is essential for studying human population dynamics and cultural behaviour^1^. It is particularly relevant for estimating the biological profile of human remains – a description of sex, age, stature, and any distinctive features, such as evidence of disease or trauma^1–5^. In bioarcheology and forensic anthropology, sex refers to the morphological and genetic traits that distinguish males and females. Typically, individuals are classified as male or female based on skeletal morphology. However, this classification may be inconclusive due to overlapping or ambiguous skeletal traits^1,2,4,6^.

Although biological sex is vital for examining demography, funerary behaviour, and epidemiology in past societies, its assessment remains challenging, particularly when remains are incomplete or fragmentary^4,7,8^. Additionally, sex estimation is usually not performed in non-adults, as it is considered unreliable before puberty, when secondary sexual characteristics become apparent^1,3,4,7,9–11^. Non-adult human skeletal remains are an important part of the archaeological record^7^ and a valuable source of information regarding the dynamics, demography, and social structure of past communities^9,12,13^. From a biocultural perspective^3^, they provide insights regarding children’s and juveniles’ lives, including mortality, growth^9^, feeding practices, and diet^12^. They can reflect potential cultural, social, and economic issues related to diseases and trauma^9^ and reveal childcare and cultural practices that may have influenced their health and well-being^3,12^. The funerary behaviours of the communities in which they grew up can also be examined, including how they were buried, the types of grave goods that accompanied them, and, if applicable, the reasons for their graves being located in different burial grounds^3,9^.

Overall, non-adult skeletons provide important insights into the lives of past communities^12,14^. However, the picture that emerges from them is limited and incomplete due to a missing puzzle piece – the estimation of biological sex. Therefore, many questions regarding the growth and lifeways of males and females remain unanswered^9^. For instance, the existence of possible sex-related differences in diet^12,15^, childcare, or burial practices^3,12,16^; even differences in mortality rates^15,17^, possibly influenced by sex-specific infanticide practices^17,18^. Although some extrapolations can be made from retrospective studies in adults, non-adults may provide a more comprehensive view of these issues^13^.

Such questions may only be answered with sex estimation of non-adults, for which methodologies began to be developed in the late 1950s^19,20^. Initial results were not particularly promising, leading to a limited number of studies conducted until the 1990s^21–28^. By the late 20th century, more reference skeletal collections became available for research, reviving interest in non-adult sex estimation^29–37^. Subsequent studies revealed early sexual dimorphism due to a neonatal hormonal surge, also known as ‘minipuberty’^38–43^. Males experience a surge in testosterone that declines to prepubertal levels within six to nine months^39,40,42,44–46^. Females undergo an oestradiol surge, with levels fluctuating for about six months before falling to prepubertal levels by age two^39,40,42,46^.

Research on biological sex estimation in infants has increased significantly over the past two decades^1^, yielding encouraging results^7,47–56^. Most studies focused on the ilium^2,57^, as it has been reported to reflect adult sex differences^58,59^. Some have employed two-dimensional (2D) and three-dimensional (3D) Geometric Morphometrics (GM) to analyse ilium features and evaluate their accuracy for subadult sex estimation^49,52,60–66^. Recently, some authors examined the morphology of the auricular surface – the anatomical region that articulates with the sacrum and is located at the upper part of the *os coxae*, the ilium^58,67^ – and how it diverges in shape and size between the sexes from an early age^6,7,54,55^.

In 2017, Luna and colleagues developed a methodology that combines qualitative morphological and morphometric analyses of the auricular surface, accurately classifying the sex of 82% of their sample^7^. In 2020, Monge Calleja and co-authors confirmed the reliability of this method by reassessing the same variables with a larger and distinct sample, resulting in findings similar to those of the original study^54^. In 2021, Luna and colleagues found this technique to be more accurate for foetuses, affirming its validity for both sexes^55^. In the same year, Marino and co-authors conducted another validation test of this method. The accuracy was lower than in previous studies. The authors tentatively suggested that this difference might be linked to interpopulation variation in the degree of sexual dimorphism^6^.

Given these encouraging results in non-adult sex estimation and their potential to enhance understanding of past populations, this research aims to test a new approach to estimate biological sex in infants aged 0 to 6 years. Using a contemporary sample with known sex and age at death, 3D GM was applied to the auricular surface to explore hypothetical morphological differences between males and females. Because of the limited data on infants’ biological sex, this estimate could be invaluable for understanding and reconstructing the paleodemography of past communities. It could also aid in exploring infant mortality rates and how they changed over time, investigating disease incidence based on biological sex, or studying the funerary treatment of infants throughout human history.

## Results

### Intra-observer error test

When analysing the Procrustes distances of the landmark sets, the highest intra-specimen Procrustes distance value is 0.039, while the lowest inter-specimen Procrustes distance value is clearly higher (0.076). Therefore, the intra-observer error is acceptable.

### Size

**Fig. 1:**
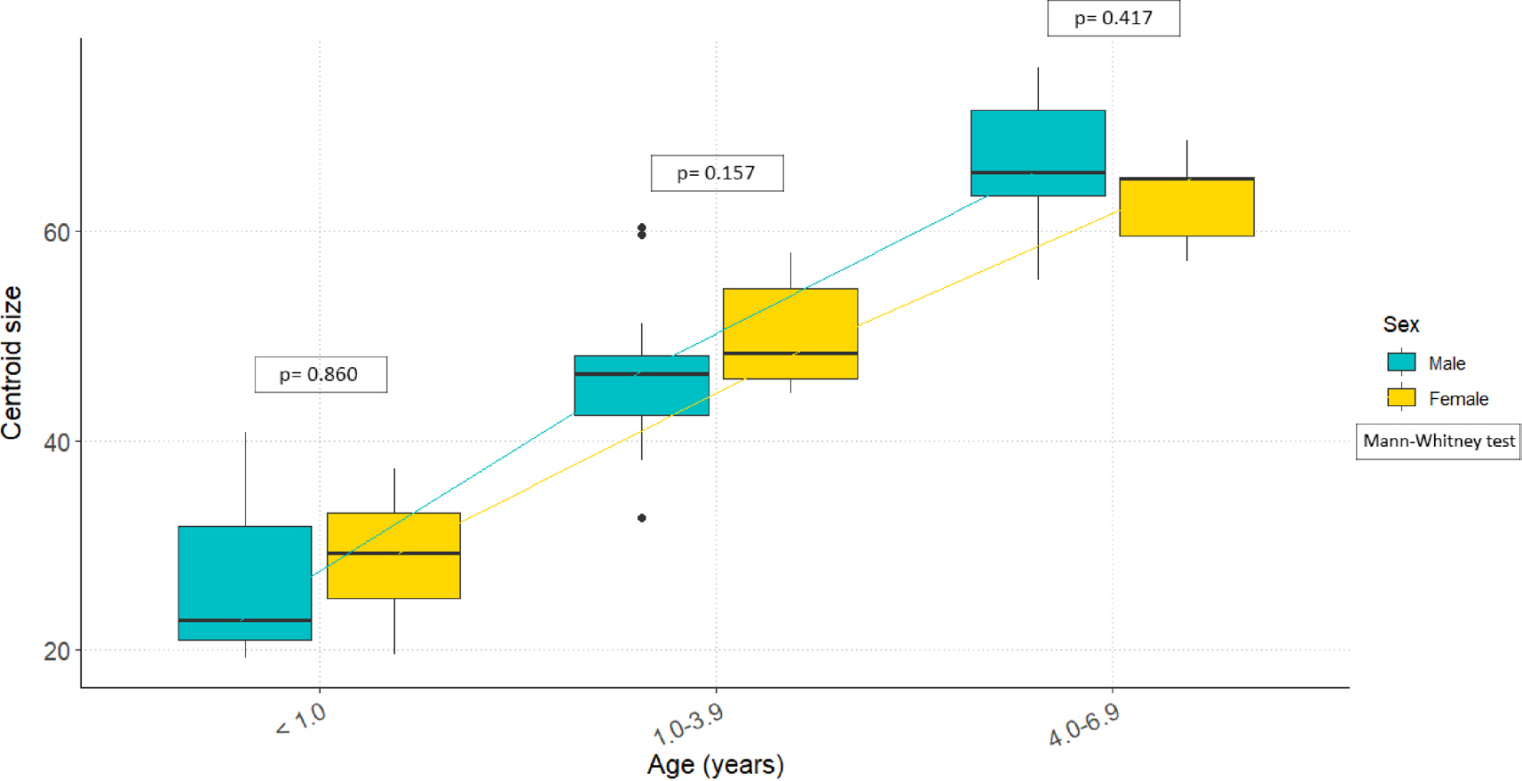
Auricular surface size (as assessed through centroid size), grouped by sex and age categories. Size increases gradually with age. Somewhat larger differences between male and female individuals are observed for the 1.0-3.9 and 4.0-6.9 age groups. The lines connect the medians of the different age categories by sex. P values for the Mann-Whitney tests comparing males and females per age cohort are also reported.

Some differences in auricular surface size were observed (Fig. 1). Despite a considerable overlap, females below 1 year of age at death show a higher median auricular surface size than males. For individuals between 1.0 and 3.9 years, females have larger auricular surfaces than males. In contrast, males exhibit larger auricular surfaces than females in the 4.0 to 6.9 years age group. Despite these observed differences, the Mann-Whitney test indicated that these variations were not statistically significant (Fig. 1).

### Shape

For the overall sample, no differences in male and female auricular surface shape were observed, as expected (see Supplementary Fig. S1). Because combining all age cohorts could obscure age-specific morphological differences, independent analyses were undertaken for each age group.

#### Individuals under one year

**Fig. 2:**
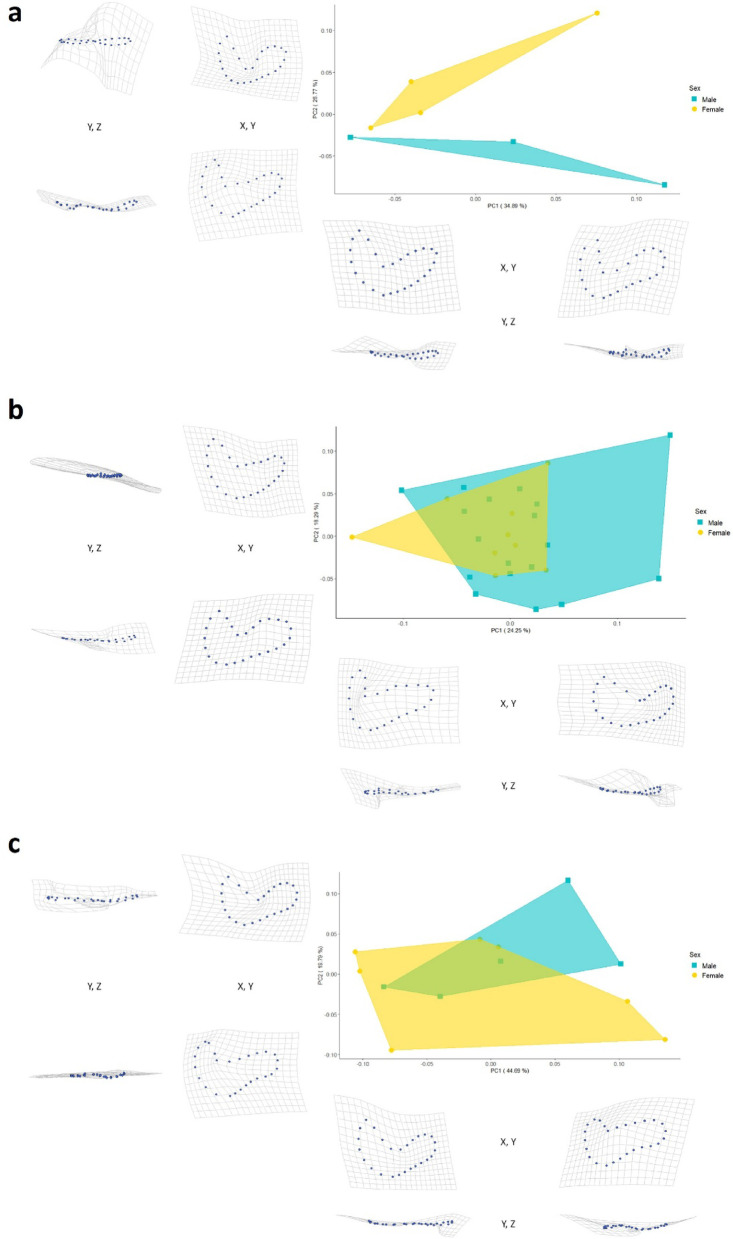
(**a**) PCA of the auricular surface shape of individuals under one year, grouped by sex. The data show no overlap between males and females along PC2, indicating differences in auricular surface morphology. (**b**) PCA of the auricular surface of the individuals aged between 1.0 and 3.9 years grouped by sex. The data indicate a nearly complete overlap of the female and male groups, suggesting no differences in auricular surface morphology. (**c**) PCA of the auricular surface shape of the individuals aged between 4.0 and 6.9 years grouped by sex. The data show a high overlap between the female and male groups, although slight differences were observed along PC2. The deformation grids on the bottom of each graph represent the auricular surface shape of the specimens closest to the minimum (on the left) and maximum (on the right) PC1 scores on the X and Y axes and the Y and Z axes. The grids on the left of each graph represent the auricular surface shape of the specimens closest to the minimum (at the bottom) and maximum (at the top) PC2 scores on the X and Y axes and the Y and Z axes. Note that the visual differences in the deformation grids are magnified by a factor of two to facilitate visualisation of the shape differences.

The Procrustes ANOVA performed on the shape coordinates, accounting for 100% of the total variance, did not reject the null hypothesis of no differences in auricular surface morphology between males and females (F = 1.23, *p* = 0.229; for further details, see Supplementary Table S1). Consistently, the PERMANOVA using the scores of the first five PCs (accounting for approximately 98% of the total variance) failed to reject the null hypothesis (Pseudo-F = 1.23, *p* = 0.229; for further details, see Supplementary Table S4). Notwithstanding the results of both tests, plotting of the PC1 and PC2 scores suggests differences in auricular surface shape distribution (Fig. 2a). Along PC1, which explains 34.89% of the total variance, individuals from both sexes overlap. In contrast, along PC2, which accounts for 26.77% of the total variance, male individuals cluster toward negative scores, while females cluster toward positive scores. Despite the visual differences observed in PC2, the Mann-Whitney test rejects the null hypothesis of no difference between the sexes, even though the PC2 p-value borders statistical significance (*p* = 0.057; see Supplementary Fig. S2).

Visualisation of shape variation along the PCs using surface warps shows that specimens with minimum PC1 scores have V-shaped auricular surfaces with equal cranial and caudal limbs. Conversely, those with maximum PC1 scores exhibit an L-shaped auricular surface with a longer caudal limb and a smaller cranial limb. Specimens near minimum PC2 scores also have an L-shaped auricular surface but feature a narrower caudal limb and a broader apex. In contrast, specimens near maximum PC2 scores display a U-shaped auricular surface with a longer cranial limb (Fig. 2a).

#### Individuals between 1.0 and 3.9 years

The results of the Procrustes ANOVA performed on the shape coordinates, which account for 100% of the total variance, reveal no sex-related morphological differences in the shape of the auricular surface (F = 0.86, *p* = 0.574; for further details, see Supplementary Table S2). The PERMANOVA results (Pseudo-F = 0.85, *p* = 0.568; for further details, see Supplementary Table S5) using the first 11 PCs, which together account for approximately 95% of the total variance, also do not support the alternative hypothesis (i.e., presence of morphological differences between sexes). Consistent with the Procrustes ANOVA and PERMANOVA results, the PCA plotting PCs 1 and 2 does not reveal differences in auricular surface shape between male and female individuals in this age group (Fig. 2b). The female group is nearly fully overlapped with the male group along PC1 (accounting for 24.25% of variance) and PC2 (accounting for 18.29% of variance). Although there appears to be greater variability among the male individuals, caution is advised, as their number is twice that of the female individuals.

Visualisation of shape variation using surface warps shows that specimens with minimum PC1 scores exhibit an L-shaped auricular surface, characterised by a short cranial limb and a long caudal limb. In contrast, those with maximum PC1 scores display a U-shaped auricular surface with nearly equal limb sizes, although the cranial limb is broader. Minimum PC2 scores correspond to a more V-shaped auricular surface with a short cranial limb and a long caudal limb. By contrast, maximum PC2 scores indicate a more U-shaped auricular surface with a longer caudal limb and a wider apex (Fig. 2b).

#### Individuals between 4.0 and 6.9 years

The results of the Procrustes ANOVA on the shape coordinates, which account for 100% of the total variance (F = 0.55, *p* = 0.831; for further details, see Supplementary Table S3), fail to reject the null hypothesis (i.e., no morphological differences between sexes). The PERMANOVA analysis of the first seven PCs, accounting for approximately 94% of the total variance, yields similar results (Pseudo-F = 0.51, *p* = 0.820; for further details, see Supplementary Table S6). Examining the distribution of specimens along PC1, which accounts for 44.69% of the total variance, and PC2, which accounts for 19.79% of the total variance, some shape differences were observed between the males and females in this age group, particularly in the PC2 scores (Fig. 2c). Notwithstanding, meaningful overlap between the groups remains, indicating no significant dissimilarities in auricular surface shape (consistent with the Procrustes ANOVA and PERMANOVA results).

Visualisation of shape variation using surface warps shows that specimens with minimum PC1 scores have a U-shaped auricular surface with limbs of nearly equal size. Conversely, those near maximum PC1 scores feature an L-shaped auricular surface with a small cranial limb and a long caudal limb. Individuals near minimum PC2 scores also display an L-shaped auricular surface, similar to those near the maximum PC1 scores. In contrast, specimens near maximum PC2 scores show a V-shaped auricular surface with limbs almost identical in size (Fig. 2c).

## Discussion

In recent decades, several studies have suggested that the auricular surface can be a reliable indicator for estimating biological sex in non-adults^7,25,29,49,54,55,61^. Given these promising findings, this study aimed to test whether it is possible to estimate the biological sex in infants by applying 3D GM to the auricular surface. The approach was designed and implemented on a contemporary sample of 46 infants with known age and sex, originating from the Lisbon Identified Skeletal Collection, also known as the Luís Lopes Skeletal Collection. Two variables were analysed: size and shape. An examination of the sample, grouped by sex and age, revealed a gradual increase in auricular surface size; however, growth rates appear to differ between males and females. For individuals under one year of age, the auricular surface size is comparable between both sexes (despite the higher median in females). From 1.0 to 3.9 years, the auricular surface is larger in females, suggesting a higher growth rate for this sex during this period. Conversely, male auricular surface size is larger from 4.0 to 6.9 years, indicating an increased growth rate for this anatomical region in males during this time span. Although there are slight distinctions in auricular surface size between the sexes, these differences are not statistically significant. Caution is necessary when interpreting the auricular surface size results for the 1.0 to 3.9 age group due to imbalanced sex representation – 33.33% females compared to 66.67% males. Sex group-size bias may be influencing results for this age group^7,54,68,69^.

Differences in auricular surface size between male and female infants have been reported previously by Wilson and collaborators (2015). They found slight differences in auricular surface size between pre-adolescent and post-adolescent males and females from the London and Lisbon Skeletal Collections, but these did not correlate with sexual dimorphism. However, in the Lisbon sample, male auricular surface size increased more significantly than that of females around the age of five^70^.

For the overall sample, no significant auricular surface shape differences were observed between males and females. When analysing the sample by age group, the PERMANOVA using the PC scores, which account for about 98% of the total variance in males and females under one year, failed to reject the null hypothesis (i.e., the absence of sex-related differences in auricular surface morphology). The Procrustes ANOVA conducted on shape coordinates to examine potential differences in 100% of the sample variance yielded similar results. From the first two principal components, which together account for 61.66% of the total variance, a clear separation of the groups is observed in PC2, with males situated near the minimum scores and females positioned along the positive scores (with no overlap between the groups). Thus, maximum differentiation is achieved between the groups, as reflected in the Mann-Whitney U statistic (U = 0, indicating that males display higher PC2 scores than females in 0 cases) and in the Area Under the Curve (AUC = 1, indicating the probability that female PC2 scores are higher than those of males). Indeed, lower U values express larger differences between the groups^71,72^, and AUC scores vary between 0 and 1, in which 1 reflects 100% probability of classification accuracy^73,74^. Last, although a Mann-Whitney test was run and the p-value reported, previous studies suggest a minimum sample size of eight individuals^75^. This sample is lower (*n* = 7), and even though the p-value of the Mann-Whitney test for PC2 is *p* = 0.057, it is the smallest achievable value (in a two-tailed test) given the available sample^72^. For individuals aged 1.0 to 3.9 years, no significant differences in auricular surface morphology were observed. Finally, male and female specimens aged 4.0 to 6.9 years displayed slight, statistically nonsignificant differences in auricular surface shape. These results indicate the possible presence of sexual dimorphism in auricular surface morphology at early ages. On the other hand, in individuals over one year old, sexual dimorphism appears to diminish, as males and females exhibit similar auricular surfaces. Alternatively, sexual dimorphism in the auricular surface may still exist, but the differences between males and females may be so subtle that this technique does not capture its morphological signal^76–78^.

Early-age trends in auricular surface morphology between the sexes may reflect the effects of ‘minipuberty’^39–43,46,54^, also referred to as the neonatal hormonal surge^38,45^. A few hours after birth, males experience a surge of testosterone^40,42,45^. Hormonal levels peak between the first and third month and then gradually decline until the sixth to ninth month of life^39,40,42,43,46^. In turn, females experience a postnatal surge of oestradiol^38,39,42,43,46^, which begins a week after birth. Hormonal levels remain elevated for approximately six months with significant fluctuations^39,40,42,43,46^. Later, between the end of the second year of life and the beginning of puberty^79^ – around 9 to 14 years in current populations^43^ – there is a period of low hormonal secretion, also designated as ‘prepubertal hiatus’^79^.

The results of this study may be related to the neonatal hormonal surge, which might explain sex-related variations in the auricular surface morphology of infants under one year old. Simultaneously, the ‘prepubertal hiatus’ may be reflected in the absence of differences in the older individuals of the sample. It is also tentatively suggested that the biomechanical forces associated with bipedal locomotion, typically reached between 12 and 15 months of age, may also influence auricular surface morphology^80^. Specifically, infants begin developing bipedal locomotion around the time when the sex differences in auricular surface shape disappear. Thus, comparable mechanical stresses and strains associated with the emergence of walking may trigger bone mechanical adaptation, leading to convergence in auricular surface shape between males and females. This aligns with authors’ assertions that morphological changes in the sacroiliac joint at the onset of locomotion are presumably attributable to mechanical forces associated with supine positioning and the transfer of body weight^81–83^. Garvin and colleagues (2021) obtained similar results when examining the ilium outline and the sciatic notch, suggesting a link to the neonatal hormonal surge. Moreover, they proposed that the lack of biomechanical forces associated with bipedal locomotion might also explain sex-related shape differences before one year of age^66^.

Monge Calleja and colleagues (2020) also discussed the neonatal hormonal surge and identified sex-related variations in the auricular surface in individuals under 19 years, with higher accuracy in those under 2 years. Specifically, the auricular surface is L-shaped in females, with a more rounded cranial arm. By contrast, the auricular surface is V-shaped in males, with a more angular cranial arm^54^. Similarly, Luna and collaborators (2021) found comparable auricular surface morphological differences between males and females under 6 years, with higher accuracy in foetuses^55^. Marino and co-authors (2021) also noted analogous sex-related variations in auricular surface morphology in individuals under 18 years of age. However, the technique was less effective for infants under one year, possibly due to the high anatomical variability of this region during the first year of life^6^.

For decades, studies have examined the reliability of the auricular surface as a sex indicator in non-adults, yielding inconsistent results. Weaver (1980) reported sexual dimorphism in the auricular surface of infants under one year, with females exhibiting a raised auricular surface and males showing no such elevation^25^. His promising findings led Hunt (1990) to test Weaver’s methodology, but the outcomes suggested that these differences in auricular surface morphology were age-related^28^. Mittler and Sheridan (1992) echoed Weaver’s observations, noting higher accuracy in children aged 9 or older^29^. Conversely, Sutter (2003) dismissed the elevation of the auricular surface as a sexually dimorphic feature in individuals under 16 years^37^.

García-Mancuso and González (2013) found sex-related differences in the auricular surface of infants under five months, with the region being narrower in males and broader in females^63^. However, in 2018, García-Mancuso and colleagues concluded that the auricular surface is not a reliable indicator for sex estimation in subadults, failing to replicate the sex-related differences observed in their earlier study^52^. Wilson and collaborators (2008) used the same criterion and successfully distinguished between males and females under 9 years old^61^. Subsequently, Wilson and colleagues (2011) classified the auricular surface as a poor sex indicator in non-adults younger than 8 years, also applying the same criterion^62^.

Despite the varying outcomes in these studies, several key aspects must be considered. The sample sizes, age distributions, and sex proportions differ significantly across studies. The populations sampled were also geographically, temporally, and culturally diverse^77,78^. Additionally, the techniques employed were diverse, with some studies combining conventional morphometrics and qualitative assessment of auricular surface morphological features^6,7,54,55^, while others focused solely on auricular surface morphology^25,28,29,37,61,62,84^ or analysed the partial outline of the auricular surface through 2D GM^49,52,63^. Consequently, it is unsurprising that findings on sexual dimorphism expression in these samples vary in both degree and pattern^77,78^.

This study is the first to use 3D GM to analyse the complete outline of the auricular surface for estimating sex in non-adults. This non-invasive, non-destructive technique uses virtual replicas – 3D models digitally reconstructed from dry bone specimens^78^ – thereby preserving the integrity of the original sample^85–88^. Although scanning technology can be expensive, costs have significantly decreased in recent years. Surface scanners are now more affordable and portable than computed tomography (CT) or micro-CT, enabling rapid acquisition of minimally processed 3D data^78,86–88^.

3D models facilitate the utilisation of GM to quantify skeletal variations that traditional morphometrics and other morphological techniques may overlook^78^. By integrating both landmark-based and semilandmark techniques, which are less prone to subjectivity and observer bias, quantitative data on the morphology of the auricular surface were gathered^89,90^. The inclusion of sliding semilandmarks enabled circumventing the limitations of relying solely on conventional landmarks, providing a more precise quantitative representation of the auricular surface^89,91,92^. Overall, this investigation, alongside previous studies^64–66,93^, demonstrates that 3D GM can be an effective tool for researching sex estimation in infants.

When interpreting the results of this study, it is important to consider an important limitation: the sample size. A total of 46 specimens were sampled from the Lisbon Identified Skeletal Collection, all of which were under 7 years old. All individuals in the collection were screened, and 57 were initially selected; however, 11 specimens were excluded due to the poor preservation of the auricular surface. The overall sex ratio of the selected sample indicates a slight predominance of males: 26 males (56.52%) compared to 20 females (43.48%). An examination of individuals by sex and age reveals an imbalanced distribution. Most individuals fall into the 1.0-3.9 years age group, comprising 27 of the 46 (58.70%). Meanwhile, only seven individuals are in the < 1.0-year group (15.22%), and 12 are in the 4.0-6.9-year age group (26.07%). Therefore, the sample available presents clear limitations that may impact this study’s results, which should be considered cautiously. This includes the results of the cohort of individuals under one year of age at death, for which no statistically significant differences were found between females and males at α = 0.05, despite a clear distinction between the groups (see details above).

Despite the sample limitations of this study, numerous previous studies examining biological sex estimation in non-adult individuals face comparable challenges due to the underrepresentation of well-preserved sub-adults, particularly infants, in documented skeletal collections^10,78,94–96^. For example, the Lisbon Identified Skeletal Collection, the largest in Portugal and the one used in this study, contains only 13.16% of individuals under 20 years old (92 out of 699 specimens)^97^. The Coimbra Identified Skeletal Collection contains no sub-adults under 7 years old^95,98^, whereas the Coimbra 21st Century Collection includes only adults over 28 years old^99,100^. The Évora Collection consists of just 3.37% of subadults (7 out of 208)^101^. This low representation is reflected in studies that have developed non-adult sex estimation techniques^68,95,101,102^. For instance, Luna and colleagues (2017) used the Coimbra Identified Skeletal Collection to develop their sex estimation method, but could sample only 34 individuals aged 7–18 years (21 females and 13 males)^7^. Monge Calleja and co-authors (2020) tested this method on 61 individuals under 19 years old from the Lisbon Collection, with only 23 (37.71%) fitting the present study’s age range^54^. Additionally, Wilson et al. (2008) evaluated 25 infants aged 9 years old or younger (17 males and eight females) from the London Collection^61^. Despite these constraints, the sample used in this study is, to the authors’ best knowledge, one of the largest assembled thus far in any comparable study.

Inter-observer error studies should also take place if different observers undertake landmarking. This is because the auricular surface lacks type I landmarks, thus rendering landmarking less straightforward. Specifically, at times, the auricular surface outline was unclear, requiring digital photography to confirm the location of the landmarks. Although the intra-observer error results showed negligible differences, supporting the reliability of the proposed landmarking protocol, testing of scoring differences across observers (i.e., inter-observer error tests) should be assessed whenever relevant.

It is also important to note that only one region of the ilium, the auricular surface, was analysed. The choice was influenced by the positive outcomes of the method proposed by Luna and colleagues (2017)^7^, and subsequent tests^54,55^. However, it is essential to recognise that other features of the ilium, such as the sciatic notch, have also been proven to be reliable sex indicators in non-adults^20,31,34,37,61–63,103^. Some researchers argue that combining multiple features improves the accuracy of sex estimation. For instance, Wilson and collaborators (2011) found that evaluating the auricular surface, sciatic notch, and iliac crest together yielded the best results^62^. Similarly, Estévez and co-authors (2017) reported increased accuracy when analysing both the sciatic notch and the auricular surface together^49^. Future studies should consider assessing multiple skeletal features to improve reliability.

This study represents the first effort to use 3D GM to assess the complete outline of the auricular surface and its potential for estimating biological sex in non-adults under 7 years old. Results are promising and point to the possible presence of sexual dimorphism in auricular surface morphology at early ages. In individuals over the age of one, differences in auricular surface morphology appear to diminish. However, it is essential to note that, at this point, non-adult sex estimation cannot be performed using this approach. The next steps should include increasing the sample size, testing other reference samples, and conducting cross-validation and cross-application tests. Additionally, more skeletal features ought to be included in the study. This would allow comparisons of outcomes across different iliac features and the evaluation of whether sex estimation accuracy increases when more than one skeletal trait is used. Finally, Artificial Intelligence (AI) techniques should also be considered for future studies. Some investigations have already assessed the performance of these techniques for sex estimation in non-adults^96^ and adults^104–109^, with very promising results.

## Methods

### Sample

According to Baccino and co-authors (2013), the terms ‘subadult’, ‘non-adult’ and ‘immature’ refer to individuals aged up to 20 years. This category includes foetuses (less than nine gestational months), newborns, infants (0–6 years), children (7–12 years) and adolescents (13–20 years). For this investigation, the focus will be on infants^110^.

Forty-six individuals, aged 0 to 6 years – 26 males (56.52%) and 20 females (43.48%) – were selected from the Lisbon Identified Skeletal Collection, currently housed at the National Museum of Natural History and Science (MUHNAC) in Portugal^97^. Access to the Collection sample was authorised by the housing institution following a formal access request and evaluation by the relevant board. All regulations of the housing institution regarding access to the sample were observed.

Individuals whose ilium was either absent or severely damaged post-mortem were excluded from the selection. Preference was given to the left ilium for analysis; however, the right ilium was used if the left was unavailable or insufficiently preserved.

**Fig. 3:**
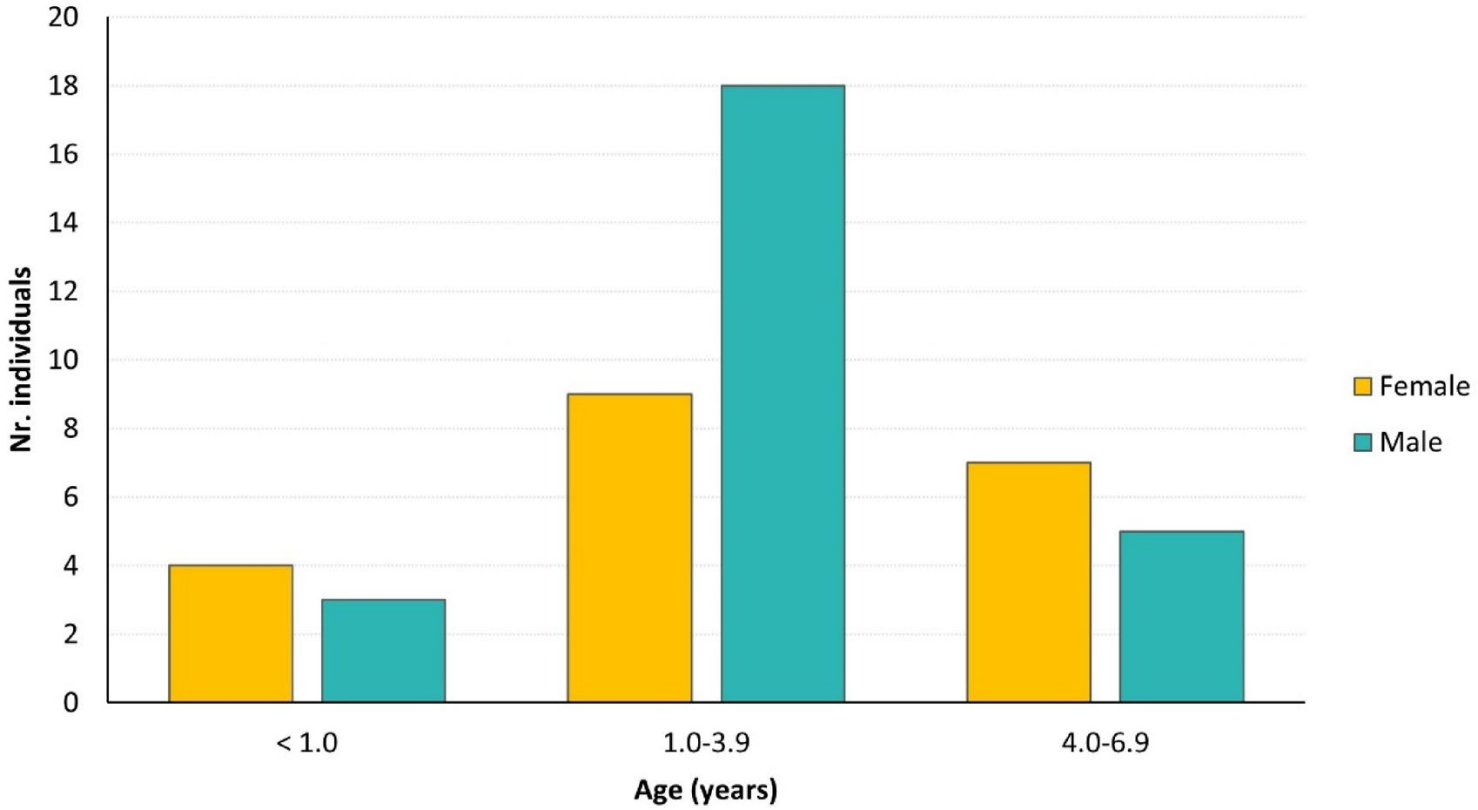
Number of individuals analysed from the Lisbon Identified Skeletal Collection, categorised by sex and age groups.

The specimens were divided into three age groups (Fig. 3) based on specific criteria: under 1.0 years, corresponding to the neonatal hormonal surge; 1.0-3.9 years; and 4.0-6.9 years, representing equal age intervals of three years. The latter age group aligns with the approximate age at which the first permanent tooth erupts^111^.

### 3D models acquisition

To follow the GM methodology^91,112,113^, it was first necessary to acquire 3D models of all the sampled specimens. Digitisation was undertaken using a portable structured-light 3D surface scanner, specifically the EinScan Pro 2X Plus model (Shinning 3D^®^). Before scanning, all specimens were photographed, and measurements of the maximum iliac length and width were taken using a sliding calliper, following the specifications outlined by Fazekas and Kósa (1978)^24^. After calibrating the scanner, it was set on a tripod in front of an automated turntable in a room with controlled lighting. The 3D models were generated using the Einscan EXScan Pro software.

### Landmarking and data collection

The collection of landmark coordinates (X, Y, and Z) from 3D models of the ilia aimed to capture the auricular surface morphology^113^. Using Viewbox version 4.1.2.1 (dHAL Software^®^, 2020), the surface of a left ilium was selected as a model to create a template. After loading the image into the software, the bone was oriented anatomically in a medial view, with the iliac crest facing upward and the greater sciatic notch facing downward. Next, landmarks were placed on the auricular surface at homologous anatomical points^89,92,114–117^. Since the area of the ilium under study lacks distinct features (Type I landmarks), the extremes of curvature were used as reference points (Type II landmarks)^91,92,116,118^. Based on previous studies that employed GM to analyse the auricular surface^49,52,119–121^, four landmarks were selected and manually placed clockwise, as indicated in Table 1 (Fig. 4).

Due to the reduced number of conventional landmarks, the analysis included the use of sliding semilandmarks along the outline of the auricular surface^91,92,114–116,122^. Initially, four curves were added manually to the template, with each curve starting and ending at a conventional landmark. The number of semilandmarks for each curve was determined based on two criteria: the mean curve length, measured across three specimens, and the spacing between points, set at approximately 3 mm. To ensure the points were equidistant, they were automatically resampled for even spacing. As a result, a total of 22 semilandmarks were added to the template, divided as indicated in Table 2.

The template was completed after establishing the landmarking sequence for the 26 points – four conventional landmarks and 22 semilandmarks (Fig. 4). Utilising it as a landmarking protocol, the landmarks and the curve semilandmarks were subsequently positioned in each specimen following the same order. For specimens with ambiguous outlines of the auricular surface, digital photography was used to locate the landmarks accurately.

The semilandmarks were automatically arranged equidistantly along the curves, and their positions were adjusted (slid) according to the reference specimen. This process was achieved by minimizing the bending energy. This procedure ensures geometrical correspondence of the semilandmarks, rendering their positioning comparable^89,92,115,116,123^. Finally, all specimen information and landmark coordinates were compiled into a Morphologika file in a standard text (.txt) file^124,125^. Considering that the reference specimen used as a template was a left ilium, and that all specimens must be from the same side, the sets of coordinates collected from right specimens were mirrored^113^.

**Table 1:** Set of landmarks (LM) used in this study for collecting coordinates from the auricular surface.

# LM	Landmark definition
1	Medial point of maximum projection at the caudal limb.
2	Point of maximum flexion at the intersection between the cranial and the caudal limb.
3	Superior point of maximum projection at the cranial limb.
4	Inferior point of maximum projection at the intersection between the cranial and the caudal limb.

**Table 2:** Description of each curve used in the outline of the auricular surface, along with the corresponding number of semilandmarks (SML).

# C	Description	Nr. of SLM
1	Curve between LM1 and LM2.	6
2	Curve between LM2 and LM3.	2
3	Curve between LM3 and LM4.	6
4	Curve between LM4 and LM1.	8

**Fig. 4:**
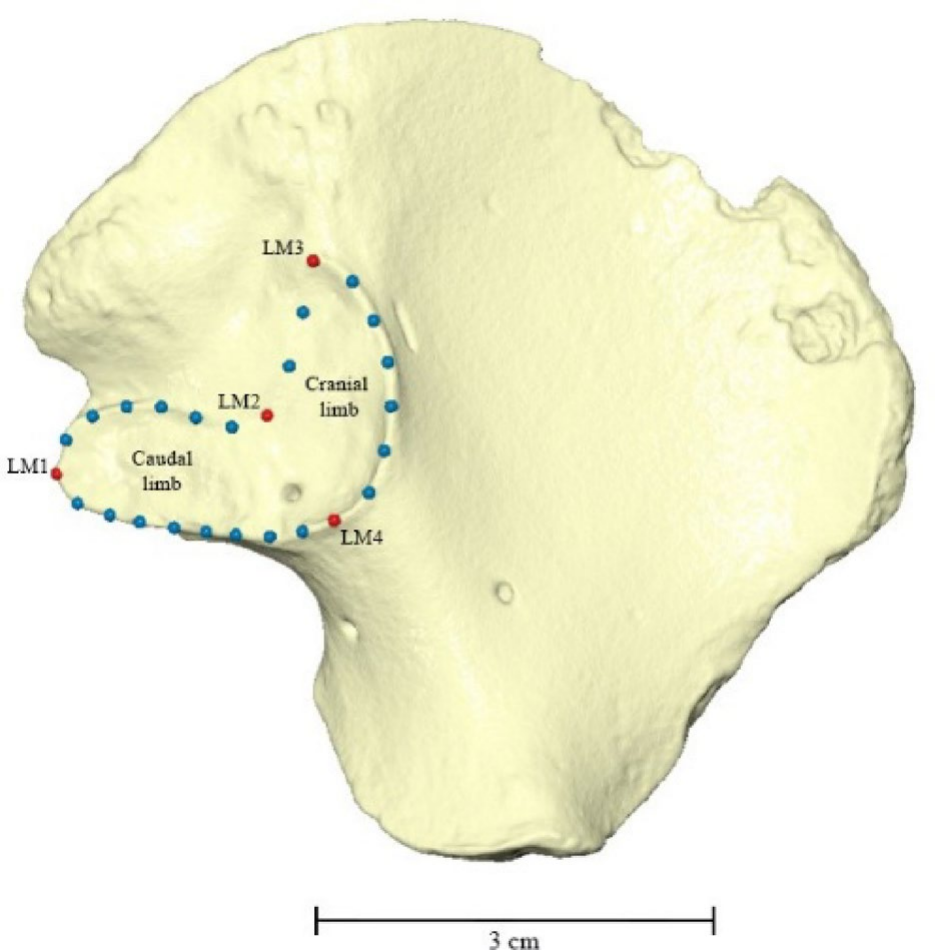
Image of the left ilium used as the model for the Viewbox template. The red dots in the image represent the conventional landmarks (4). The blue dots indicate the sliding semilandmarks (22), evenly distributed across the four curves along the outline of the auricular surface.

### Morphological analysis

The Geomorph package, version 4.0.8^126^, designed for standard GM analyses of 2D and 3D landmark data in the open-source software R (version 4.4.0), was used for all data analysis^127^.

Initially, the Morphologika file was imported into R, and a Generalised Procrustes Analysis (GPA) was performed. This analysis superimposes the landmark configurations of all specimens. By removing size, orientation, and location as variables, the raw coordinates were transformed into Procrustes shape coordinates^89,113,115,117,118^. The standard geometric morphometrics size measure for landmark configurations is the centroid size^89,117,128^. Therefore, this variable was used to compare the sizes of the auricular surface across the specimens.

To investigate shape (e.g., geometric properties of an object) variance in the auricular surface, a Principal Component Analysis (PCA) was performed on the shape coordinates, which reduced dimensionality. The resulting Principal Components (PC) scores represent the variability within the dataset^89^, with the first Principal Component accounting for the largest portion of this variability^128^. The shape variance associated with each relevant PC was then visualised using thin-plate splines (TPS)^91,114^.

Subsequently, statistical tests were performed to explore potential differences between groups. Nonparametric tests are recommended for small sample sizes^75,129,130^. Therefore, potential size differences in the auricular surface related to sexual dimorphism were assessed using Mann-Whitney tests. To investigate sex-related differences in auricular surface shape, a PERMANOVA (Permutational Multivariate Analysis of Variance) was conducted using all PC scores, which explain approximately 95% of the shape variance. Furthermore, a Procrustes ANOVA was performed on the Procrustes shape coordinates to assess potential shape differences in the auricular surface between sexes, accounting for 100% of the shape variance. When relevant, the Mann-Whitney test was also employed to compare specific PC scores to assess differences in male and female auricular surface morphology.

### Intra-observer error test

To assess the level of variation introduced by the observer during data collection (i.e., landmarking), a measurement error test was conducted involving the repeated measurement of the same specimen^131^ on a subset of the reference sample. Seven left specimens were selected. The same set of landmarks was acquired from each ilium three times independently, with each observation being performed at least two days apart. All data were compiled into a Morphologika file, which was then imported into R. A GPA was performed on the raw landmarks collected, and pairwise Procrustes distances representing shape differences between paired sets of shape landmarks configurations^89,132^ were analysed to determine if the intraobserver error is negligible. Following established criteria from previous studies^133,134^, intraobserver error is acceptable if the largest Procrustes distance among the repeated measures (intra-individual distances) is lower than the smallest Procrustes distance among all other individuals (inter-individual distances).

## Supplementary Information

Below is the link to the electronic supplementary material.

Supplementary Material 1

## Data Availability

The data that support this study are available from the corresponding author upon reasonable request.
