# Supplementary material for: A geometric morphometrics approach to sex estimation of infants from 0 to 6 years using the auricular surface
Source: Sci Rep. 2026 Feb 28;16:11422. doi: 10.1038/s41598-026-35321-y (PMC13057032; doi:10.1038/s41598-026-35321-y)
Supplement: Supplementary file 1 — Supplementary Material 1 [file 41598_2026_35321_MOESM1_ESM.pdf]

# A geometric morphometrics approach to sex estimation of infants from 0 to 6 years using the auricular surface

**Supplementary Data**

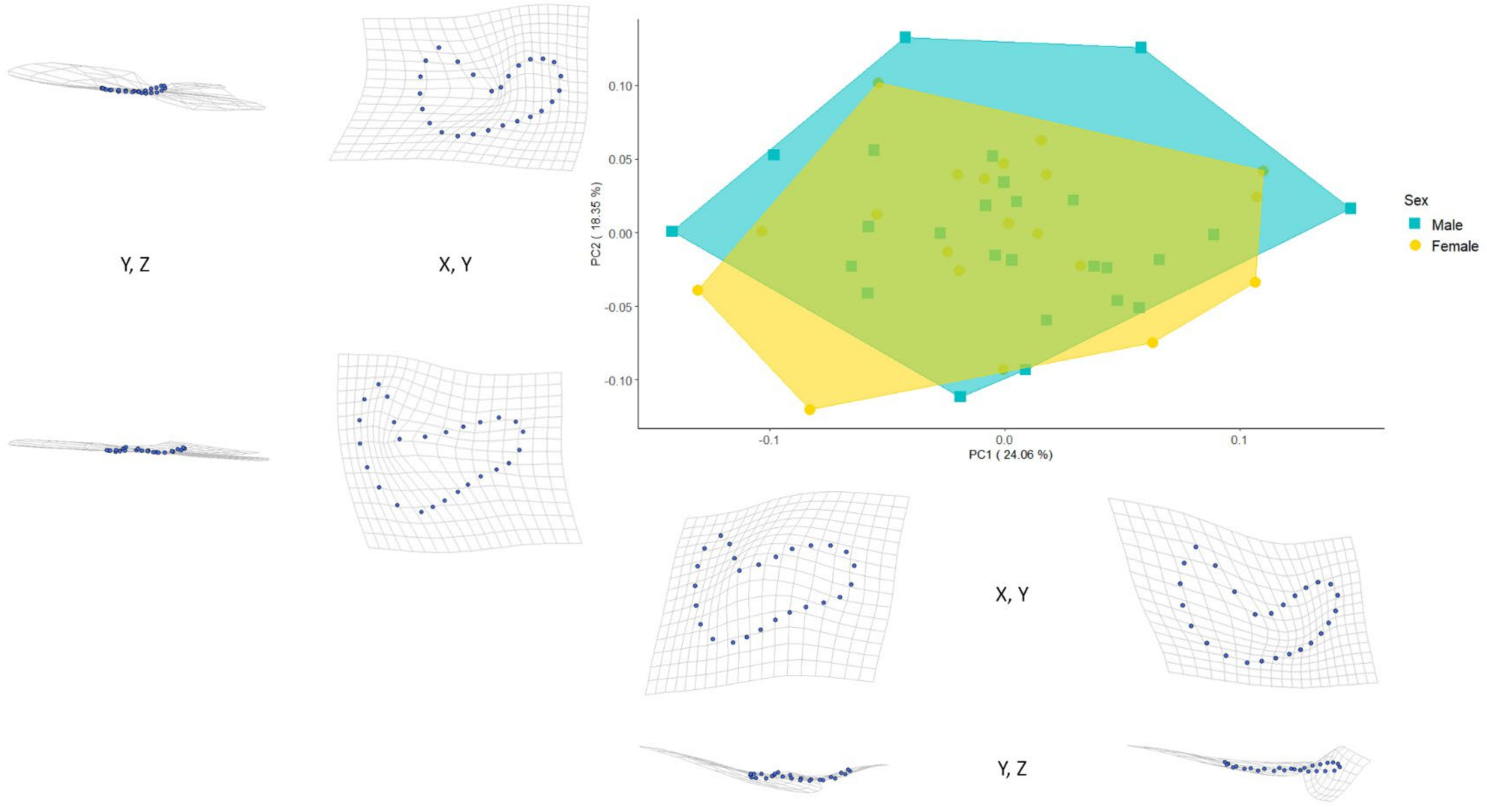


**Supplementary Figure S1.** PCA of the auricular surface shape, with individuals grouped by sex. The data show a substantial overlap between the male and female groups, indicating no discernible morphological differences between them. The deformation grids on the bottom of the graph represent the auricular surface shape of the specimens closest to the minimum (on the left) and maximum (on the right) PC1 scores on the X and Y axes and the Y and Z axes. The grids on the left of the graph represent the auricular surface shape of the specimens closest to the minimum (at the bottom) and maximum (at the top) PC2 scores on the X and Y axes and the Y and Z axes. To facilitate shape differences visualisation, the deformation grids are magnified by a factor of two.

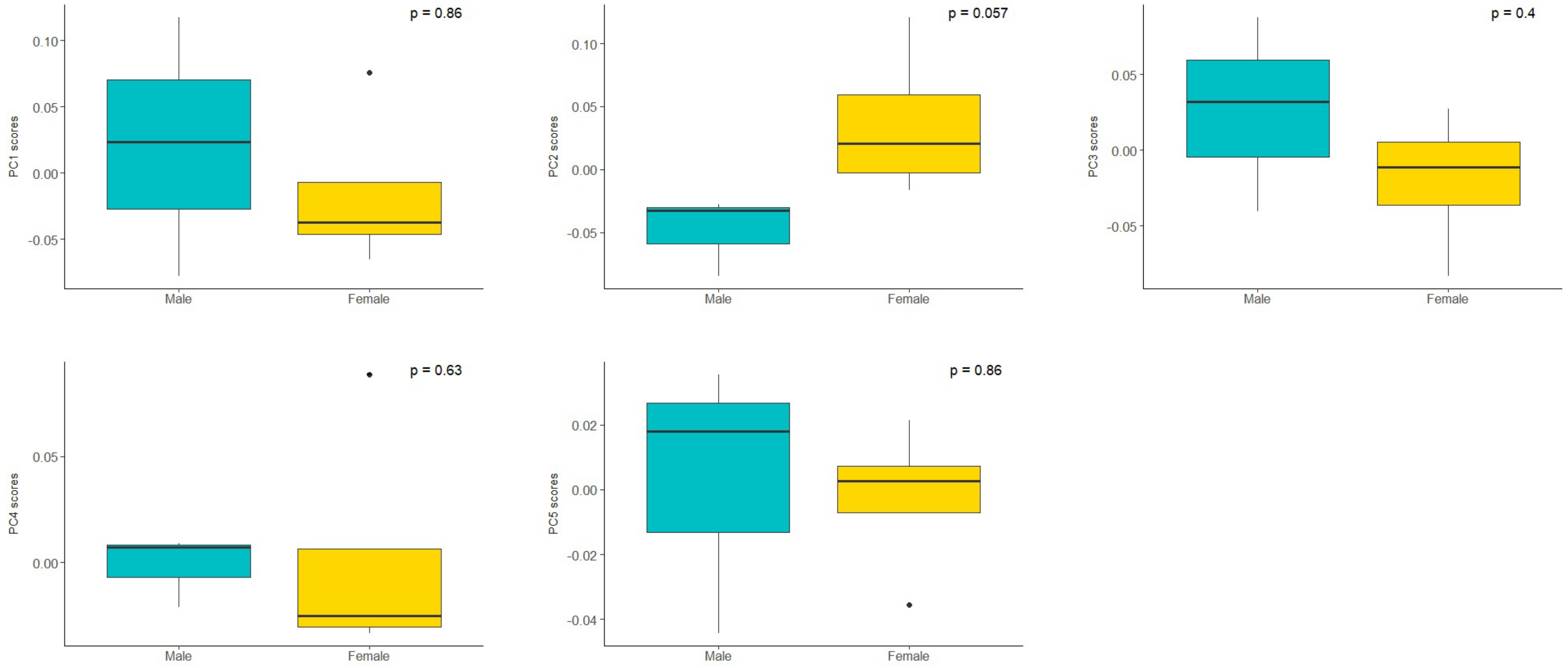


**Supplementary Figure S2.** Distribution of the principal component scores explaining approximately 98% of the total variance in the auricular surface shape of infants under one year, categorised by sex. The results of the Mann-Whitney tests conducted for each PC score are shown in the top-right corner of the boxplots. Notably, there is overlap between the two groups, except for PC2. The p-value for this principal component is very close to the threshold for statistical significance ($p = 0.057$).

**Supplementary Table S1.** Procrustes ANOVA (with 999 permutations at an α = 0.05) results for individuals under one year (SS = Sum of Squares).

| Source | *df* | SS | $R^2$ | F | *p*-value |
|---|---|---|---|---|---|
| **Sex** | 1 | 0.01878 | 0.198 | 1.23 | 0.229 |
| **Residuals** | 5 | 0.07606 | 0.802 | | |
| **Total** | 6 | 0.09484 | 1.000 | | |

**Supplementary Table S2.** Procrustes ANOVA (with 999 permutations at an α = 0.05) results for individuals between 1.0 and 3.9 years (SS = Sum of Squares).

| Source | *df* | SS | $R^2$ | F | *p*-value |
|---|---|---|---|---|---|
| **Sex** | 1 | 0.01273 | 0.03316 | 0.86 | 0.574 |
| **Residuals** | 25 | 0.37115 | 0.96684 | | |
| **Total** | 26 | 0.383888 | 1.00000 | | |

**Supplementary Table S3.** Procrustes ANOVA (with 999 permutations at an α = 0.05) results for individuals between 4.0 and 6.9 years (SS = Sum of Squares).

| Source | *df* | SS | $R^2$ | F | *p*-value |
|---|---|---|---|---|---|
| **Sex** | 1 | 0.009343 | 0.05205 | 0.5491 | 0.831 |
| **Residuals** | 10 | 0.170143 | 0.94795 | | |
| **Total** | 11 | 0.179486 | 1.00000 | | |

**Supplementary Table S4.** PERMANOVA (using a Euclidean distance matrix with 9999 permutations at an α = 0.05) results for individuals under one year (SS = Sum of Squares).

| Source | *df* | SS | $R^2$ | Pseudo-F | *p*-value |
|---|---|---|---|---|---|
| **Sex** | 1 | 0.01834 | 0.19725 | 1.23 | 0.229 |
| **Residual** | 5 | 0.07465 | 0.80275 | | |
| **Total** | 6 | 0.09299 | 1.00000 | | |

**Supplementary Table S5.** PERMANOVA (using a Euclidean distance matrix with 9999 permutations at an $\alpha = 0.05$) results for individuals between 1.0 and 3.9 years (SS = Sum of Squares).

| Source | *df* | SS | $R^2$ | Pseudo-F | *p*-value |
|---|---|---|---|---|---|
| **Sex** | 1 | 0.01199 | 0.03277 | 0.85 | 0.568 |
| **Residual** | 25 | 0.35397 | 0.96723 | | |
| **Total** | 26 | 0.36597 | 1.00000 | | |

**Supplementary Table S6.** PERMANOVA (using a Euclidean distance matrix with 9999 permutations at an $\alpha = 0.05$) results for individuals between 4.0 and 6.9 years (SS = Sum of Squares).

| Source | *df* | SS | $R^2$ | Pseudo-F | *p*-value |
|---|---|---|---|---|---|
| **Sex** | 1 | 0.008163 | 0.04847 | 0.5094 | 0.820 |
| **Residual** | 10 | 0.160266 | 0.95153 | | |
| **Total** | 11 | 0.168429 | 1.00000 | | |
